# Effect of malarial infection on haematological parameters in population near Thailand-Myanmar border

**DOI:** 10.1186/1475-2875-13-218

**Published:** 2014-06-05

**Authors:** Manas Kotepui, Bhukdee Phunphuech, Nuoil Phiwklam, Chaowanee Chupeerach, Suwit Duangmano

**Affiliations:** 1 Medical Technology Programme, School of Allied Health Sciences and Public Health, Walailak University, Nakhon Si Thammarat 80161, Thailand; 2 Medical Technology Laboratory, Phop Phra Hospital, Phop Phra District, Tak 63160, Thailand; 3 Institute of Nutrition, Mahidol University, Nakhon Pathom 73170, Thailand; 4 Department of Medical Technology, Faculty of Associated Medical Sciences, Chiang Mai University, Chiang Mai 50200, Thailand

**Keywords:** Complete blood count, Phob Phra, Malaria infection, Thrombocytopenia

## Abstract

**Background:**

Malaria is a major mosquito-borne public health problem in Thailand with varied haematological consequences. The study sought to elucidate the haematological changes in people who suspected malaria infection and their possible predictive values of malaria infection.

**Methods:**

Haematological parameters of 4,985 patients, including 703 malaria-infected and 4,282 non-malaria infected, who admitted at Phop Phra Hospital, Tak Province, an area of malaria endemic transmission in Thailand during 2009 were evaluated.

**Results:**

The following parameters were significantly lower in malaria-infected patients; red blood cells (RBCs) count, haemoglobin (Hb), platelets count, white blood cells (WBCs) count, neutrophil, monocyte, lymphocyte and eosinophil counts, while mean corpuscular volume (MCV), mean corpuscular haemoglobin (MCH), Mean corpuscular haemoglobin concentration (MCHC), neutrophil-lymphocyte ratio (NLR), and monocyte-lymphocyte ratio (MLR) were higher in comparison to non-malaria infected patients. Patients with platelet counts < 150,000/uL were 31.8 times (odds ratio) more likely to have a malaria infection. Thrombocytopenia was present in 84.9% of malaria-infected patients and was independent of age, gender and nationality (P value < 0.0001).

**Conclusion:**

Patients infected with malaria exhibited important changes in most of haematological parameters with low platelet, WBCs, and lymphocyte counts being the most important predictors of malaria infection. When used in combination with other clinical and microscopy methods, these parameters could improve malaria diagnosis and treatment.

## Background

Changes in haematological parameters are likely to be influenced by any disease condition including endemic diseases, such as malaria, that can affects health of mankind with various clinical presentations. Malaria is a major cause of deaths in the tropical area of the world. Two hundred and nineteen million cases were reported worldwide in 2010 [1]. Haematological changes are some of the most common complications in malaria and they play a major role in malaria pathogenesis. These changes involve the major cell types such as RBCs, leucocytes and thrombocytes [2-5]. Malaria infected patients tended to have significantly lower platelets, WBCs, lymphocytes, eosinophils, RBCs and Hb level, while monocyte and neutrophil counts were significantly higher in comparison to non-malaria infected patients [2-4,6-8]. One study showed patients with higher WBCs count compared with community controls [9]. The most common complication during malaria infection is thrombocytopenia [6,10-12]. Persons with platelet counts < 150,000/μL were 12-15 times more likely to have malaria infection than persons with platelet counts > 150,000/μL [6]. A previous study found that the ratio of monocytes to lymphocytes correlated with risk of clinical malaria during follow-up [5]. In Thailand, about five million people are in the area of high malaria transmission mostly in border between Thailand and Burma [13]. Clinical diagnosis is widely used for diagnosis of malaria especially in these areas.

Fever and other signs and symptoms are known to be sensitive measures of malaria infection but they lack specificity and positive predictive values especially in areas where malaria is less prevalent [3,14] and it may be difficult to distinguish the sign and symptom of disease from other viral or bacterial infections [15]. Typically, microscopic slide examination of peripheral blood remains the most widely used test and is the gold standard for detecting malaria infection [16]. However, due to it requires technical expertise and is time-consuming in smear examinations. Haematological changes during malaria infection, such as thrombocytopenia and leucocytosis or leucopenia are well recognized. Diagnostic value of these haematological alterations may be easily obtained and useful in people living in malaria endemic areas.

Phop Phra is a district (Amphoe) in the southwestern part of Tak Province, northern Thailand. It is in part of the Thailand-Myanmar border and is the most common destination for ethnic minorities from Myanmar who migrate into Thailand for agricultural work. This province has the highest number of malaria cases for 10 consecutive years. The present study aim to examine the occurrences of haematological changes and their diagnostic values in people infected with malaria in Phop Phra Hospital. Haematological parameters including WBCs, RBCs, platelets, red cell distribution width (RDW), MCV, MCH, MCHC, and Hb level of people infected with malaria were compared with uninfected people.

## Methods

Data collection used in this study was approved by The Phob Phra Hospital and The Ethical Clearance Committee on Human Rights Related to Researches Involving Human Subjects of Walailak University. The data were collected from the Medical Technology Laboratory Unit, Phob Phra Hospital, Tak Province during January to December 2009. Laboratory records of people suspected with malaria infection such as fever, other signs and symptoms as medical doctor recommended were reviewed. Four thousand nine hundred and eighty five patients with full data recorded were included in this analysis. This included 703 malaria and 4,282 non-malaria cases. The data were retrieved from the venous blood sample drawn into EDTA tubes for preparation of the thick and thin smears and automated for determination of Complete Blood Counts (CBCs). Blood counts were performed using BC-5200 Haematology Analyzer (Mindray, Nanshan, Shenzhen, China). The Analyzer provided data on WBCs, RBCs, Hb level, platelet counts, MCV, MCH, MCHC, RDW and five part differentials. The Haematology Analyzer have an internal quality control (IQC) two-times daily (First time in the morning before running sample and second time in the afternoon) and also external quality control (EQC) three times per year with Department of Medical sciences. Blood slides were prepared and stained with Giemsa. Microscopic abnormality of blood in smear and presence or absence of malaria were determined, the species and the number of asexual parasites. Data analysis was performed using SPSS ver. 11.5 (SPSS Inc., Chicago, IL, USA). Normally distributed of continuous data were determined using The Kolmogorov-Smirnov Test. Comparing between 3 groups of continuous data was using a One-way ANOVA, whereas data not conforming to normal distribution were compared by a Kruskal-Wallis Test. Categorical data were compared using Pearson Chi-Square Test. Association between two continuous data was assessed by Pearson Correlation, whereas data not conforming to normal distribution were compared by Spearman’s rank correlation. Diagnostic accuracy of haematological parameters was measured by calculating sensitivity, specificity, predictive values and odds ratios and 95% confidence intervals.

## Results

Four thousand nine hundred and eighty five patients were included in this study. The median age was 26 years (18-40) and 52.4% were males. Seven hundred and three (n = 4,985) of the subjects had malaria infection confirmed by microscopy while the remaining were non-infection and were used as controls. In malaria-infected patients, three hundred and fifty-two (50.1%) had *Plasmodium falciparum* infection, whereas three hundred and fifty-one (49.9%) had *Plasmodium vivax* infection. There were significant differences between age, gender, and nationality of patients with group of patients (P value < 0.0001). Malaria positive patients were Burma 50.5%, followed by Thai 49.2% and other nationalities 0.3%, respectively (Table 1).

**Table 1:** General characteristics

	***Plasmodium falciparum***	***Plasmodium vivax***	**Non-malaria**	**P value**
	***n = 352***	***n = 351***	**n = 4,282**	
Demographic				
Age, medium (IQR)	26 (18-40)	23 (16-36)	16 (7-35)	<0.0001*
Male/female, n (%)	215 (61.1)/137 (38.9)	209 (59.5)/142 (40.5)	2,190 (51.1)/2,092 (48.9)	<0.0001**
Thai/Burma/Others, n (%)	160 (45.5)/192 (54.5)	186 (53)/165 (47)	3,230 (75.4)/1,052 (24.6)	<0.0001**

*Comparison of 3 groups using The Kruskal Wallis Test, **Comparison of 3 groups using Pearson Chi-Square, IQR = interquartile range.

The median values of haematological parameters of falciparum malaria, vivax malaria and non-malaria infected group were compared using Kruskal-Wallis Test due to non-parametric distribution of data. Median values of RBCs, platelets, WBCs, and all absolute leukocyte components counts were significantly lower in patients with falciparum malaria compared to those with vivax malaria and non-malaria infected groups. Conversely, median of MCV, MCH, MCHC, NL ratio, and ML ratio were significantly higher in falciparum malaria than those with vivax malaria and non-malaria infected groups. A median value of Hb was lower in patients with falciparum malaria compared to those with vivax malaria and non-malaria infected groups. Monocyte and eosinophil counts were higher in patients with vivax malaria compared to those with falciparum malaria and non-malaria infected groups. There was no significant difference in median of RDW between three groups (Table 2).

**Table 2:** Haematological values in study population

**Variable**	***Plasmodium falciparum***	***Plasmodium vivax***	**Non-malaria**	P value*
	**Median (IQR)**	**Median (IQR)**	**Median (IQR)**	
RBC (x10^6^/μL)	4.33 (3.78–4.8)	4.45 (4.04–4.88)	4.63 (4.25–5.02)	<0.0001
Haemoglobin (g/dL)	11.7 (9.8–13.1)	11.9 (10.4–13)	11.8 (10.5–13.1)	0.041
MCV (fL)	83.45 (78–3–87.7)	82.2 (75–86.8)	81.7 (74.6–87.3)	0.001
MCH (pg/cell)	27 (25–28.9)	26.9 (24–28.7)	26.3 (23.5–28.6)	<0.0001
MCHC (g/dL)	32.6 (31.6–33.4)	32.5 (31.7–33.3)	26.3 (23.5–28.6)	<0.0001
RDW (%)	12.8 (12.1–13.9)	12.8 (12.1–13.9)	12.7 (12–14)	0.967
Platelet (×10^3^/μL)	72.5 (44–112)	89 (63–133)	242 (181–317)	<0.0001
WBC (×10^3^/μL)	5.76 (4.52–7.99)	5.59 (4.48–7.18)	8.71 (6.17–12.4)	<0.0001
Neutrophil (×10^3^/μL)	3.82 (2.77–5.18)	3.44 (2.63–4.51)	5.08 (3.23–8.04)	<0.0001
Lymphocyte (×10^3^/μL)	1.28 (0.83–1.98)	1.31 (0.9–1.89)	2.29 (1.55–3.41)	<0.0001
Monocyte (×10^3^/μL)	0.4 (0.25–0.61)	0.4 (0.26–0.6)	0.57 (0.38–0.84)	<0.0001
Eosinophil (×10^3^/μL)	0.17 (0.73–0.31)	0.2 (0.08–0.37)	0.24 (0.12–0.47)	<0.0001
Basophil (×10^3^/μL)	0.05 (0.04–0.06)	0.06 (0.04–0.07)	0.08 (0.06–0.11)	<0.0001
NL ratio	2.8 (2.81–4.88)	2.67 (1.69–4.22)	2.17 (1.2–3.95)	<0.0001
ML ratio	0.3 (0.3–0.43)	0.29 (0.19–0.47)	0.24 (0.17–0.38)	<0.0001

*Comparison of 3 groups using The Kruskal Wallis Test.

### Red blood cells

Anaemia was defined as Hb level <11g/dl for both males and females based on the WHO cutoff value. The median of Hb in patients with falciparum malaria (11.7 g/dL) was significantly lower than those with vivax malaria (11.9 g/dL) and non-malaria (11.8 g/dL) groups (P value = 0.041). One hundred and thirty-four (38.1%) of patients with falciparum malaria; one hundred and fifteen (32.8%) of patients with vivax malaria; one thousand three hundred and sixty-five (31.9%) of patients with non-malaria groups had anaemia. There was no significant association between status of malaria infection and Hb cut-off (P value = 0.057). The median of RBCs count was significantly lower in patients with falciparum malaria (4.33 × 10^6^/μL) than non-falciparum malaria (4.45 × 10^6^/μL) and non-malaria (4.63 × 10^6^/μL) groups (P value <0.0001). Patients with RBCs count less than 4 × 10^6^/μL was frequently seen in patients with falciparum malaria (32.4%) compared to those with vivax malaria (22.2%) and non-malaria (13.6%) groups (P value < 0.0001). The median of MCV, MCH, and MCHC in both patient with falciparum malaria and vivax malaria were significantly higher than those with non-malaria group (P value < 0.001). There was no significant association between status of malaria infection and MCV > 100fL, MCH > 32pg/cell, and MCHC >37g/dL cutoff value (P value > 0.05).

### White blood cells

Leucopenia was defined as total WBCs count < 4,000/μL. There was significant difference in total WBCs count between three groups of patients (P value < 0.0001). Leukocyte components were also significantly affected. Neutrophil, lymphocyte, monocytes, eosinophil and basophil counts were all significantly decreased in patients with falciparum malaria and vivax malaria as compared to those with non-malaria group (P value < 0.0001). Neutrophil count > 2,800/μL was frequently seen in the malaria-infected group (27.9%) than the non-malaria infected group (19.5%) (P value < 0.0001). Lymphocytes < 800 /μL (lymphocytopenia) was frequently seen in the malaria-infected group (20.1%) than the non- infected group (4.6%) (P value < 0.0001). There were significant differences in NL ratio and ML ratio between three groups of patients (P value < 0.0001). High NL ratio (>2.8) was frequently seen in the malaria-infected group than the non-infected group (P value < 0.0001). High ML ratio (>0.25) was frequently seen in the malaria-infected group than the non-infected group (P value < 0.0001).

### Platelets

Thrombocytopenia was defined as platelets count < 150,000/μL. There were 597 patients with Thrombocytopenia (84.9%) in the malaria-infected group. The median of platelet count in patients with falciparum malaria (72.5 × 10^3^/μL) and vivax malaria (89 × 10^3^/μL) were significantly lower than those with non-malaria (242 × 10^3^/μL) group (P value < 0.0001). Thrombocytopenia was also associated with anaemia in the malaria group (r = -0.107, P value = 0.004) and age (r = -0.235, P value < 0.0001).

### Diagnostic values of haematological parameters

Most of haematological parameters had good specificity but lack of sensitivity to detect infection of malaria parasite. However, low platelet count with platelet count < 150,000/uL had high sensitivity (85%) and specificity (85%) for diagnosis of malaria infection. This parameter had better odds ratios. Patients with platelets count < 150,000/uL were up to 31.8 (OR) times more likely to have malaria infection than those with normal platelets count (P value < 0.0001, OR = 31.8, CI = 25.1-39.7) (Table 3). It could be predict that the association between thrombocytopenia and malaria infection would not be significant following adjustment for age, gender, and nationality of patients. Age, gender, and nationality, correlated with risk of malaria infection (Table 4). However, adjusting for these demographic variables did not confound the association between thrombocytopenia and malaria infection.

**Table 3:** Sensitivity, specificity, predictive value and odds ratio of the haematological parameters in diagnosis of Malaria

**Variable**	Sensitivity* (95% CI)	Specificity* (95% CI)	PPV* (95% CI)	NPV* (95% CI)	**OR (95% CI)**	**PLR**	**NLR**
RBC <4×10^6^/μL	27 (24–31)	86 (85–87)	25 (22–28)	88 (87–89)	2.4 (2–2.9)	2	0.8
Hb < 11 g/dL	35 (32–39)	68 (67–70)	15 (14–17)	87 (85–88)	1.2 (1–1.4)	1.1	0.9
MCV >100 fL	1 (0.5–2)	98 (97–99)	10 (4–19)	86 (85–87)	0.7 (0.3–1.4)	0.7	1
MCH >32 pg/cell	3 (2–5)	97 (96–97)	13 (8–19)	86 (85–87)	0.9 (0.6–1.4)	0.9	1
MCHC >37 g/dL	2 (1–4)	98 (98–99)	18 (11–28)	86 (85–87)	1.4 (0.8–2.4)	1.4	1
Platelets <150,000/μL	85 (82–87)	85 (84–86)	48 (45–51)	97 (96–98)	31.8 (25.1–39.7)	5.6	0.2
WBCs <4,000/μL	17 (14–20)	94 (93–94)	30 (26–35)	88 (87–89)	3 (2.4–3.7)	2.7	0.9
Neutrophils <2,800/μL	35 (32–39)	69 (68–70)	16 (14–17)	87 (86–89)	1.2 (1.0–1.4)	1.1	0.9
Lymphocytes <800/μL	25 (23–28)	78 (77–79)	17 (15–19)	86 (85–87)	1.4 (1.1–1.6)	1.2	1
Monocytes <80/μL	3 (2–5)	98 (97–99)	19 (12–26)	86 (85–87)	1.4 (0.9–2.2)	1.4	1
Eosinophils <40/μL	27 (24–30)	71 (69–72)	13 (11–15)	86 (84–87)	0.9 (0.8–1)	0.9	1
Basophils <16/μL	89 (86–91)	12 (11–13)	14 (13–15)	86 (83–89)	1.0 (0.8–1.3)	1	1
NL ratio >2.8	49 (45–52)	61 (60–63)	17 (15–19)	88 (87–89)	1.5 (1.3–1.8)	1	0.8
ML ratio >0.25	58 (54–62)	54 (53–56)	17 (16–19)	89 (88–90)	1.7 (1.4–1.9)	1.3	0.8

*PPV*, positive predictive value; *NPV*, negative predictive value; *PLR*, positive likelihood ratio; *NLR*, negative likelihood ratio, *Percent, *OR*, odds ratio.

**Table 4:** The association between risk of malaria and platelet count is independent of age, gender and nationality

**Analysis type**	**Variable**	**Malaria positive/negative**	
		B*	**P value**
Univariate	Platelets <150,000	0.024	<0.0001
	Age	−0.01	<0.0001
	Gender	0.373	<0.0001
	Nationality	−1.153	<0.0001
Multivariate	Platelets <150,000	0.024	<0.0001
	Age	0.014	<0.0001
	Gender	−0.115	0.287
	Nationality	0.935	<0.0001
	Constant	−2.926	<0.0001

*unstandardized variables.

## Discussion

This study confirms that haematological abnormalities in malaria infection are common. Leucopenia was frequently seen in the malaria-infected patients which was confirmed by other studies that have demonstrated leucopenia [6,15] and contrast with another study that had demonstrate leucocytosis [3]. Neutrophil and lymphocyte counts were the most important leukocytic changes associated with malaria infection. The decrease in lymphocyte counts associated with malaria observed in this study may due to reflect redistribution of lymphocytes with sequestration in the spleen [6,17]. The decrease in neutrophil count associated with malaria infection was in contrast with previous studies which suggested that it might be activated neutrophil production or release from the marrow or suppressed peripheral removal [3].

The observation of decreased monocytes counts in this study was also in contrast with previous studies [3,18]. Mononuclear cells, which are activated by Plasmodium during malarial infection, produce inflammatory cytokines, such as tumor necrosis factor (TNF), interleukin-1 (IL1) and interleukin-6 (IL6). These cytokines stimulate the hepatic synthesis of acute phase inflammatory proteins, including CRP, which increase during malaria infection [17].

Anaemia is one of the most common complications in malaria infection especially in younger children and pregnant women in high transmission areas [19]. The pathogenesis of anaemia during malaria infection is not clearly understood. However, It is thought to result from the parasite’s primary target is the red blood cell resulting in RBCs destruction, accelerated removal of both parasitized and non-parasitized [20], bone marrow dysfunction [21] and the level of parasitemia [22]. This study, reported a significant reduction of Hb level and RBC count whereas MCV, MCH, and MCHC level in patients infected with malaria were higher as compared to non-malaria infected group. There are no evidence relate to higher level of MCV, MCH, and MCHC and pathogenesis of malaria infection but it has been suggested that people with α^+^-thalassaemia protected malaria infection by a direct interaction between the parasite (*Plasmodium falciparum*) and the altered thalassaemic erythrocyte, resulting in reduced parasite load [23]. Most studies showed that α^+^-thalassaemia homozygotes have better protection against severe malarial anaemia compared to heterozygotes [24,25]. Moreover, patients with microcytosis and higher erythrocyte count associated with α^+^-thalassaemia homozygosity had an advantage against severe malarial anaemia during acute infection with *Plasmodium falciparum*[26].

Platelet abnormalities in malaria are both qualitative and quantitative change. In this study, platelet counts were significantly reduced in malaria-infected people. Thrombocytopenia occurred in 84.9% of malaria-infected patients. These observations may imply that thrombocytopenia may be a marker of Plasmodium infection. The association of platelet count and malaria infection has previously been described [3,7,18]. Patients with thrombocytopenia were also likely to have an anaemia (r = -0.107, P value = 0.004) and correlate with age (r = -0.235, P value < 0.0001) as previously reported from a study in Kenya and Nigeria [3,8]. There were various hypotheses about thrombocytopenia occurring in malaria infection. Thrombocytopenia seems to occur through peripheral destruction [9], excessive removal of platelets by spleen pooling [27,28] as well as platelet consumption by the process of disseminated intravascular coagulopathy (DIC) [29]. Adequate or increased number of megakaryocytes in the bone marrow affects decreased thrombopoiesis an unlikely cause of thrombocytopaenia in malaria [27]. Immune-mediated destruction of circulating platelets has been postulated as a cause of thrombocytopenia seen in malaria infection. Platelets have also been shown to mediate clumping of *P. falciparum* infected erythrocytes [30]. This could lead to pseudo thrombocytopenia. Malaria-infected patients have elevated levels of specific immunoglobulinG (IgG) in the blood which binds to platelet-bound malaria antigens possibly leading to accelerated destruction of platelets [10]. The previous study revealed that platelet aggregation, which is the platelet clumps are falsely counted as single platelet by the analyzers thus causing pseudo-thrombocytopenia [3]. Additionally, during malaria infection, endothelial activation was activated and may contribute to loss of barrier function of the endothelium and organ dysfunction. This process may use platelets and their released proteins as an important regulator of endothelial permeability resulting in thrombocytopaenia [31].

In this study, the sensitivity, specificity, positive predictive value, negative predictive value and diagnostic accuracy for all haematoparameter were determined. Among of those parameter, thrombocytopenia (platelet counts < 150,000/μl) had the best sensitivity, specificity and negative predictive value (85% sensitivity, 85% specificity, and 97% negative predictive value) which were higher than the previous studies [11,12] while the positive predictive value was low (48%). The two most reliable haematological parameters for predicting malaria in people from endemic areas were thrombocytopenia and leucopenia. Patients with thrombocytopenia were 31.8 times more likely to have malaria infection than those with normal platelets count. Thrombocytopenia and leucopenia had sensitivity of 85% and 17%, specificity 85% and 94%, respectively to predict malaria infection. Patients with leucopenia (WBCs <4,000/μL) were 2.7 times more likely to have malaria infection than those with normal leucocytes count. High neutrophil counts, leucopenia, and lymphopenia had good specificity but lacked sensitivity to screening of malaria infection. The monocytes to lymphocytes ratio (ML ratio) in this study was high supported by the previous study that the ML ratio, which was measured in peripheral blood was directly correlates with risk of clinical malaria during follow-up [5]. This may due to the major role of the monocytes in the first time of innate immune response by releasing IFNγ, to response to malaria infection [32]. When considered the neutrophil to lymphocytes ratio (NL ratio) in this study, it was also high which supported that the NL ratio was found to correlate with malaria parasitaemia [4].

Limitations of this study included lack of previous medical histories such as other diseases that may have analysis bias such as Hb diseases, anaemia, bacteria or virus infection, which could potentially affect the interpretation of the results.

## Conclusions

In conclusion, the association of haematological parameters and a diagnosis of malaria infection among people living in malaria endemic area was retrieved. The most commonly changed parameters were platelet count, Hb, RBC, MCV, MCH, MCHC, WBC, neutrophil, and lymphocyte counts. Presence of thrombocytopenia in people from endemic areas may be useful as supportive diagnostic criteria for malaria in case with low level of parasite number. Therefore, when used with other clinical and microscopy parameters, it can significantly improve malaria diagnosis and timely further treat for malaria infection. The large sample size used in this study gives this analysis power to detect differences between malaria infected and non-malaria infected people.

## Competing interests

The authors declare that they have no conflict of interest.

## Authors’ contributions

MK participated in study design, data analysis, statistical analysis, and manuscript writing and. BP and NP participated in data collection including CBC and malaria microscopy. SD and CC participated in data analysis and manuscript writing. All authors read and approved the final manuscript.

## References

[B1] WHO World malaria report 2012 2012 Geneva: World Health Organization

[B2] Bakhubaira S Hematological parameters in severe complicated *Plasmodium falciparum* malaria among adults in Aden Turk J Haematol 2013 30 394 399 10.4274/Tjh.2012.0086 24385830 PMC3874965

[B3] Maina RN Walsh D Gaddy C Hongo G Waitumbi J Otieno L Jones D Ogutu BR Impact of *Plasmodium falciparum* infection on haematological parameters in children living in Western Kenya Malar J 2010 9 Suppl 3 S4 10.1186/1475-2875-9-S3-S4 21144084 PMC3002140

[B4] van Wolfswinkel ME Vliegenthart-Jongbloed K De Mendonca Melo M Wever PC McCall MB Koelewijn R Van Hellemond JJ Van Genderen PJ Predictive value of lymphocytopenia and the neutrophil-lymphocyte count ratio for severe imported malaria Malar J 2013 12 101 10.1186/1475-2875-12-101 23506136 PMC3608093

[B5] Warimwe GM Murungi LM Kamuyu G Nyangweso GM Wambua J Naranbhai V Fletcher HA Hill AV Bejon P Osier FH Marsh K The ratio of monocytes to lymphocytes in peripheral blood correlates with increased susceptibility to clinical malaria in Kenyan children PLoS One 2013 8 e57320 10.1371/journal.pone.0057320 23437368 PMC3577721

[B6] Erhart LM Yingyuen K Chuanak N Buathong N Laoboonchai A Miller RS Meshnick SR Gasser RA Jr Wongsrichanalai C Hematologic and clinical indices of malaria in a semi-immune population of western Thailand Am J Trop Med Hyg 2004 70 8 14 14971691

[B7] Gerardin P Rogier C Ka AS Jouvencel P Brousse V Imbert P Prognostic value of thrombocytopenia in African children with falciparum malaria Am J Trop Med Hyg 2002 66 686 691 12224575 10.4269/ajtmh.2002.66.686

[B8] Adedapo AD Falade CO Kotila RT Ademowo GO Age as a risk factor for thrombocytopenia and anaemia in children treated for acute uncomplicated falciparum malaria J Vector Borne Dis 2007 44 266 271 18092534

[B9] Ladhani S Lowe B Cole AO Kowuondo K Newton CR Changes in white blood cells and platelets in children with falciparum malaria: relationship to disease outcome Br J Haematol 2002 119 839 847 10.1046/j.1365-2141.2002.03904.x 12437669

[B10] Moulin F Lesage F Legros AH Maroga C Moussavou A Guyon P Marc E Gendrel D Thrombocytopenia and *Plasmodium falciparum* malaria in children with different exposures Arch Dis Child 2003 88 540 541 10.1136/adc.88.6.540 12765928 PMC1763122

[B11] Shiraz Jamal Khan YA Mumtaz Ali M Thrombocytopenia as an Indicator of Malaria in Adult Population Malar Res Treat 2012 405981 1 4 10.1155/2012/405981 PMC3395156 22811952

[B12] Mahmood A Yasir M Thrombocytopenia: a predictor of malaria among febrile patients in Liberia Infect Dis J 2008 14 41 44

[B13] WHO World Malaria Report 2013 2013 Geneva: World Health Organization

[B14] WHO New perspective, malaria diagnosis 2000 Geneva: World Health Organization

[B15] Lathia TB Joshi R Can hematological parameters discriminate malaria from nonmalarious acute febrile illness in the tropics? Indian J Med Sci 2004 58 239 244 15226575

[B16] WHO World Malaria Report 2010 2011 Geneva: World Health Organization

[B17] Wickramasinghe SN Abdalla SH Blood and bone marrow changes in malaria Baillieres Best Pract Res Clin Haematol 2000 13 277 299 10.1053/beha.1999.0072 10942626

[B18] Abdalla SH Peripheral blood and bone marrow leucocytes in Gambian children with malaria: numerical changes and evaluation of phagocytosis Ann Trop Paediatr 1988 8 250 258 2467614 10.1080/02724936.1988.11748582

[B19] Menendez C Fleming AF Alonso PL Malaria-related anaemia Parasitol Today 2000 16 469 476 10.1016/S0169-4758(00)01774-9 11063857

[B20] Price RN Simpson JA Nosten F Luxemburger C Hkirjaroen L ter Kuile F Chongsuphajaisiddhi T White NJ Factors contributing to anemia after uncomplicated falciparum malaria Am J Trop Med Hyg 2001 65 614 622 11716124 10.4269/ajtmh.2001.65.614 PMC4337986

[B21] Abdalla SH Hematopoiesis in human malaria Blood Cells 1990 16 401 416 discussion 417-409 2257320

[B22] Kitua AY Smith TA Alonso PL Urassa H Masanja H Kimario J Tanner M The role of low level *Plasmodium falciparum* parasitaemia in anaemia among infants living in an area of intense and perennial transmission Trop Med Int Health 1997 2 325 333 9171840 10.1111/j.1365-3156.1997.tb00147.x

[B23] Nagel RL Roth EF Jr Malaria and red cell genetic defects Blood 1989 74 1213 1221 2669996

[B24] Williams TN Wambua S Uyoga S Macharia A Mwacharo JK Newton CR Maitland K Both heterozygous and homozygous alpha + thalassemias protect against severe and fatal *Plasmodium falciparum* malaria on the coast of Kenya Blood 2005 106 368 371 10.1182/blood-2005-01-0313 15769889

[B25] May J Evans JA Timmann C Ehmen C Busch W Thye T Agbenyega T Horstmann RD Hemoglobin variants and disease manifestations in severe falciparum malaria JAMA 2007 297 2220 2226 10.1001/jama.297.20.2220 17519411

[B26] Fowkes FJ Allen SJ Allen A Alpers MP Weatherall DJ Day KP Increased microerythrocyte count in homozygous alpha(+)-thalassaemia contributes to protection against severe malarial anaemia PLoS Med 2008 5 e56 10.1371/journal.pmed.0050056 18351796 PMC2267813

[B27] Beale PJ Cormack JD Oldrey TB Thrombocytopenia in malaria with immunoglobulin (IgM) changes BMJ 1972 1 345 349 10.1136/bmj.1.5796.345 5008661 PMC1787235

[B28] Skudowitz RB Katz J Lurie A Levin J Metz J Mechanisms of thrombocytopenia in malignant tertian malaria BMJ 1973 2 515 518 10.1136/bmj.2.5865.515 4714466 PMC1589556

[B29] Essien EM The circulating platelet in acute malaria infection Br J Haematol 1989 72 589 590 10.1111/j.1365-2141.1989.tb04329.x 2673332

[B30] Pain A Ferguson DJ Kai O Urban BC Lowe B Marsh K Roberts DJ Platelet-mediated clumping of P*lasmodium falciparum*-infected erythrocytes is a common adhesive phenotype and is associated with severe malaria Proc Natl Acad Sci U S A 2001 98 1805 1810 10.1073/pnas.98.4.1805 11172032 PMC29338

[B31] Brouwers J Noviyanti R Fijnheer R de Groot PG Trianty L Mudaliana S Roest M Syafruddin D van der Ven A de Mast Q Platelet activation determines angiopoietin-1 and VEGF levels in malaria: implications for their use as biomarkers PLoS One 2013 8 e64850 10.1371/journal.pone.0064850 23755151 PMC3670845

[B32] Rhee MS Akanmori BD Waterfall M Riley EM Changes in cytokine production associated with acquired immunity to *Plasmodium falciparum* malaria Clin Exp Immunol 2001 126 503 510 10.1046/j.1365-2249.2001.01681.x 11737069 PMC1906215

